# Optimising pulmonary rehabilitation for α_1_-antitrypsin deficiency: a qualitative study of patient and clinician perspectives

**DOI:** 10.1183/23120541.00332-2025

**Published:** 2026-03-09

**Authors:** Fawaz A. Alwadani, Rachel Adams, Mohammed Alshahrani, Harriet Pittaway, Anita Pye, Alice M. Turner

**Affiliations:** 1 Department of Applied Health Research, School of Health Sciences, University of Birmingham, Birmingham, UK; 2 Department of Physical Therapy, College of Nursing and Allied Health Sciences, Jazan University, Jazan, Saudi Arabia; 3 Health Services Management Centre, University of Birmingham, Birmingham, UK; 4 Institute of Inflammation and Ageing, University of Birmingham, Birmingham, UK

## Abstract

**Background:**

α_1_-antitrypsin deficiency (AATD) is a rare genetic condition that predisposes individuals to early-onset respiratory disease. While pulmonary rehabilitation (PR) improves function and quality of life in COPD, limited evidence exists on its suitability and optimisation for AATD, which presents distinct clinical and psychosocial challenges.

**Methods:**

This qualitative study (November 2023–July 2024) explored how PR services can be adapted to better meet the needs of individuals with AATD. Semi-structured interviews were conducted with 14 patients. 10 healthcare professionals participated in the study: eight took part in three online focus groups and two were interviewed individually. Data were analysed using the Framework Method to identify key improvement areas. A critical-to-quality diagram was developed to translate insights into service-level recommendations.

**Results:**

Six key themes emerged: 1) accessibility and customisation, 2) personalised rehabilitation care, 3) integrated diagnosis and referral pathways, 4) emotional and social support, 5) post-rehabilitation support and 6) technology integration. Participants identified the need for flexible, locally delivered or hybrid PR models, tailored exercise prescriptions (*e.g.*, high-intensity interval training), earlier referrals and condition-specific peer and digital support.

**Conclusion:**

Tailored PR models for AATD should address disease-specific needs through flexible delivery formats, personalised approaches to desaturation management and structured referral and follow-up pathways. These findings provide a roadmap for optimising PR in AATD and may inform improvements in rehabilitation for other early-onset or rare respiratory conditions.

## Shareable abstract

**Tailored pulmonary rehabilitation for α_1_-ATD is vital. Flexible, patient-centred programmes including personalised exercise, streamlined referrals and structured follow-up can overcome α_1_-ATD's unique challenges and improve long–term outcomes.** https://bit.ly/4fPG2DR

## Introduction

α_1_-antitrypsin deficiency (AATD) is a rare, inherited condition associated with early-onset emphysema and COPD, leading to accelerated lung function decline, frequent exacerbations and reduced quality of life [1–4]. Compared with smoking-related COPD, AATD typically presents earlier and progresses more rapidly, necessitating tailored clinical and rehabilitative strategies [2, 5, 6].

Pulmonary rehabilitation (PR) is a cornerstone of care for individuals with chronic lung diseases, with well-established benefits including improved exercise capacity, reduced dyspnoea and enhanced quality of life [4, 7–10]. PR also contributes to a reduced number of hospitalisations and increased long-term physical activity in COPD populations. However, most PR programmes have been developed for the general COPD population and may not adequately address the unique clinical and psychosocial needs of people with AATD [11–14].

Distinct features of AATD such as earlier disease onset, exertional oxygen desaturation and exercise intolerance may necessitate adaptations to standard PR content and delivery models [3, 8, 11, 13]. High-intensity interval training (HIIT) has been suggested as a strategy to improve tolerance and performance in patients who desaturate with exertion, although evidence for its effectiveness in AATD remains limited [10, 13, 15]. The role of supplemental oxygen during PR is also debated due to mixed evidence, stigma and practical barriers to use [14, 16, 17]. Importantly, earlier disease onset in AATD highlights the need for sustained, flexible and personalised rehabilitation approaches that can support long-term self-management.

Despite these specific needs, research focused on PR in AATD remains scarce. Much of the existing literature is derived from broader COPD studies, which may not account for disease-specific factors such as genetic identity, social stigma and variable access to PR services [6, 11, 14, 18]. Moreover, few studies have examined how PR delivery can be adapted to better serve individuals with AATD, particularly from the perspectives of patients and healthcare professionals (HCPs).

This qualitative study explores the perspectives of individuals with AATD and HCPs involved in PR to identify priority areas for service improvement. Rather than focusing solely on barriers or facilitators, the study examines how PR can be meaningfully adapted to address AATD-specific needs across clinical, social and healthcare system levels. Findings are synthesised into a critical-to-quality (CTQ) framework to inform the design and delivery of tailored PR services for AATD, with insights that may also be transferable to other early-onset or rare respiratory conditions.

## Methods

### Study design

This qualitative study explored the lived experiences of individuals with AATD and the perspectives of HCPs involved in PR service delivery. Conducted over 7 months (November 2023–July 2024), the study followed the Standards for Reporting Qualitative Research (SRQR) guidelines [19].

The study was led by F.A.A., a physiotherapist with experience in rehabilitation and qualitative research, supported by a multidisciplinary team. Four observational visits (two hospital-based, two community-based) were conducted by F.A.A. to inform the development of interview guides and enhance contextual understanding of PR services.

### Topic guide development

Two semi-structured topic guides (supplementary material) were developed: one for patients (exploring PR experiences, perceived barriers and suggestions for improvement) and one for HCPs (focusing on PR structure, referral pathways and AATD-specific considerations). The guides were informed by a 2024 systematic review on PR in AATD [11], and refined through team discussion. The HCP guide was piloted with a senior physiotherapist and revised based on feedback.

### Recruitment and sampling

Purposive sampling was used to ensure variation in age, gender, disease severity, oxygen use and PR experiences among AATD patients, supplemented by convenience sampling [20]. Patients were eligible if they were aged ≥18 years, had a confirmed diagnosis of AATD and were willing to discuss their experiences with PR or structured exercise. This included individuals who had completed PR (n=10), as well as those who had engaged in community-based exercise, were awaiting PR or declined formal rehabilitation but remained physically active (n=4). Patients were recruited from the Centre for Rare Diseases at Queen Elizabeth Hospital Birmingham. Weekly clinics and patient records were reviewed to support sampling diversity. 11 interviews were conducted online and three in person, each lasting 42–60 min. Online sessions were arranged *via* email, while in-person interviews took place in private consultation rooms following clinic appointments.

HCPs were recruited through clinical and academic networks based on their expertise in PR service delivery and/or AATD management. Eligible participants held clinical or academic roles in physiotherapy, respiratory medicine, palliative care or related disciplines with direct relevance to rehabilitation. A total of 10 HCPs were recruited: eight participated in three online focus groups, and two took part in individual interviews. All meetings with HCPs were conducted online *via* Microsoft Teams (Microsoft Corporation, Redmond, WA, USA), with each focus group lasting approximately 90 min.

### Data collection and analysis

All interviews and focus groups were conducted *via* Microsoft Teams and audio-recorded with participant consent using the platform's built-in recording function. Transcripts were generated using Microsoft Teams’ automated transcription service, then reviewed, edited and anonymised by the lead researcher (F.A.A.) to ensure accuracy.

F.A.A. conducted all individual interviews and served as the primary moderator for all focus groups. Each focus group (FG) was co-facilitated by a designated member of the research team (R.A. (FG1), H.P. (FG2) or Kamen Dosanjih (FG3)) who supported group facilitation, observed interactional dynamics and maintained field notes. Observational notes and post-session debriefings contributed to analytic reflexivity and supported data triangulation.

Data were analysed using the seven-stage Framework Method [21]: 1) transcription, 2) familiarisation with the data, 3) coding, 4) developing a working analytical framework, 5) applying the framework, 6) charting data into a matrix and 7) interpreting the data. Initial coding was conducted using NVivo (version 14, Lumivero, Melbourne, Australia), with two researchers (F.A.A. and M.A.) independently coding the first three transcripts from both patient and HCP datasets. These datasets were initially analysed separately, and a shared analytical framework was developed through discussion. The framework was then applied consistently across all transcripts. In stage 6, data were charted into a framework matrix using Microsoft Word, and in stage 7, patient and HCP data were integrated to identify overarching themes related to barriers, facilitators and improvement priorities in PR for AATD. R.A. provided methodological oversight throughout and supported the refinement of the analytical framework.

### Development of the critical-to-quality diagram

To visually synthesise key insights, a CTQ diagram was developed to map themes to quality drivers and actionable service recommendations. The initial CTQ framework was created by the lead author (F.A.A.) using Lucidchart, based on finalised framework matrices. It was iteratively reviewed by co-authors until consensus was achieved.

While data saturation was not formally assessed, thematic saturation was inferred through repeated codes and the richness of the data. Data supporting the thematic structure are illustrated through selected quotes in the Results section.

## Results

### Participant characteristics

14 AATD patients (P1–P14) participated in the study, of whom 71.4% were male. The median age was 62 years (interquartile range (IQR) 50.0–79.0 years). Most were ex-smokers (64.3%), with key clinical characteristics including a median forced expiratory volume in 1 s (FEV_1_) of 1.49 L (58.8% predicted) and oxygen saturation of 94.0% at rest when well (IQR 84.0%–97.0%). Descriptive data extracted from patient records are summarised in table 1.

**TABLE 1:** Baseline demographic and clinical characteristics of participants with α_1_-antitrypsin deficiency (AATD)

Category	Variable	Median (IQR) or n (%)
**Demographics**	Gender	Male: 10 (71.4%); female: 4 (28.6%)
	Age (years)	62.0 (50.0–79.0)
	BMI (kg·m^−2^)	23.7 (18.3–40.0)
**Respiratory function**	FEV_1_ (L)	1.49 (0.70–3.41)
	FEV_1_ (% predicted)	58.8% (36.6%–82.5%)
	FVC (L)	3.99 (2.04–5.71)
	*K*_CO_ (mmol·min^−1^·kPa^−1^·L^−1^)	0.74 (0.24–1.63)
	O_2_ saturation (%)	94.0 (84.0–97.0)
**Oxygen therapy**	Oxygen use	Yes: 4 (28.6%) (ambulatory: 3, LTOT: 1); no: 10 (71.4%)
**Exacerbations**	Exacerbation last year	Yes: 6 (42.9%); no: 8 (57.1%)
	Exacerbation frequency (events per year)	1.0 (1.0–7.0)
**Smoking history**	Smoking status	Ex-smoker: 9 (64.3%); never smoker: 5 (35.7%)

Data are presented as median (IQR) for continuous variables and as number (%) for categorical variables. IQR: interquartile range; BMI: body mass index; FEV_1_: forced expiratory volume in 1 second; FVC: forced vital capacity; *K*_CO_: transfer coefficient of the lung for carbon monoxide; LTOT: long-term oxygen therapy.

10 HCPs contributed insights across two individual interviews and three online focus groups. Disciplines represented included cardiorespiratory physiotherapy, respiratory medicine, exercise prescription, epidemiology, primary care, palliative care, and PR service delivery. Participants held a mix of clinical and academic roles, including senior physiotherapists, clinical specialists, and professors.

### Thematic analysis

Using the Framework Method, six key themes emerged from integrated analysis of patient and HCP perspectives:

1) Accessibility and customisation
2) Personalised rehabilitation care
3) Integrated healthcare and referral pathways
4) Emotional and social support
5) Post-rehabilitation support
6) Technology integration

These themes represent priority areas for improving PR engagement, design and long-term impact for individuals with AATD.

Table 2 presents an overview aligning patient and HCP perspectives across these themes, identifying shared barriers, facilitators and service challenges. Table 3 provides illustrative quotes that reflect lived experiences and professional insights.

**TABLE 2:** Alignment of patient and healthcare professional (HCP) perspectives on pulmonary rehabilitation (PR) for α_1_-antitrypsin deficiency (AATD)

Themes	Patient perspective	HCP perspective
**Accessibility and customisation**	Travel distance and scheduling conflicts limit attendance. Many prefer community-based PR for flexibility.	PR access varies based on funding and availability. Expanding community-based and hybrid PR models could improve participation.
**Personalised rehabilitation care**	Patients favour goal-directed rehabilitation, but struggle with rapid desaturation. Some find HIIT beneficial, while oxygen use remains debated.	Goal-oriented rehabilitation is key. HIIT benefits some, but the role of oxygen therapy remains unclear. HCPs emphasise individualised assessments.
**Integrated healthcare and referral pathways**	Patients report diagnostic delays and inconsistent PR referrals, often relying on specialists rather than primary care.	Limited AATD awareness among primary care providers leads to inconsistent referrals. Standardised referral pathways and clinician education are needed.
**Emotional and social support**	Patients seek AATD-specific peer support, as generic COPD groups feel less relevant. Stigma around “genetic COPD” affects emotional well-being.	HCPs recognise the need for structured peer networks and acknowledge limited awareness of available support resources.
**Post-rehabilitation support**	Lack of structured follow-up after PR discharge makes it difficult to maintain exercise routines. Patients want ongoing community-based and digital check-ins.	Funding constraints limit long-term PR support. Community-based follow-ups and digital monitoring could sustain long-term benefits.
**Technology integration**	Hybrid PR (in person and online) improves engagement. Patients lack AATD-specific digital tools for tracking and self-management.	Hybrid PR models with structured assessments are promising. Digital tools tailored for AATD could enhance adherence and personalisation.

HIIT: high-intensity interval training.

**TABLE 3:** Summary of patient and healthcare professional (HCP) perspectives on pulmonary rehabilitation (PR) for individuals with α_1_-antitrypsin deficiency (AATD), categorised into key themes

Themes	Patient quotes	HCP quotes
**Accessibility and customisation**	“I couldn't keep up with regular times because of work, and it's hard to make appointments that fit around everything.” (P5) “It would make a big difference to have somewhere close by.” (P3) “I thought it was absolutely brilliant. The programme was very local, and it was structured well. They started by introducing us to light exercises and walking, to see how we managed our breathlessness. Then, they gradually took us into the gym to do more structured exercises. It was great that it wasn't overwhelming, and I felt it was very well done.” (P7) “Everyone there was far older than me – like 20-plus years older. I was the youngest. We all had our own strengths and limitations.” (P9)	“Patients would likely prefer to go to a centre closer to where they live rather than travel far, even if a different facility might be more advanced.” (HCP2) “We provided evening and weekend classes, and inherently, those classes were attended by younger patients.” (HCP4) “Some patients might be aware they need to exercise but are uncertain about what to do. For these patients, we sometimes refer them to Be Active services where they can engage in exercise more flexibly. Many enjoy these services because they're closer to home and offer more flexibility. We often refer patients there after rehab to keep them engaged in exercising.” (HCP3) “It's important to recognise that different service providers might approach this differently. For instance, a community physiotherapy service might provide advice and education, even if the patient isn't immediately referred to pulmonary rehab.” (HCP1)
**Personalised rehabilitation care**	“Once I start exercising, I drop right down to 80 [oxygen saturation] quite quickly.” (P9) “If more people knew what it's like, they might actually understand. My GP didn't know much about α_1_.” (P6) “I have oxygen at home… but when I go to the gym, I haven't been using it. I do things at a slower rate, and I know when to stop.” (P10) “Sometimes I have to put it on because I'm really knackered, but I only try to keep it on for a little while.” (P4) “α_1_ patients, in my opinion, should do as much as they can, and I mean as much as they can. And when you've done enough, you should try and do a bit more. There's no suitable exercise specific for α_1_, but you have to keep going.” (P8)	“We try to base our exercises on the patients' goals, making them very goal-focused.” (HCP4) “There's also the question of whether the patient would benefit from oxygen during exercise and whether they're willing to use it. Many patients are reluctant to use portable oxygen because they don't want to be seen with it, which can lead to them doing less activity. The use of oxygen in exercise requires a lot of discussion and education with the patient.” (HCP2) “For individuals who desaturate or have poor exercise tolerance, high-intensity interval aerobic training is a useful way of prescribing aerobic exercise.” (HCP5) “A large proportion of the time, they're very similar – they have chronic lung diseases, severe breathlessness and emphysema. However, they typically present at a younger age than you would expect for COPD.” (HCP3)
**Integrated healthcare and referral pathways**	“If I could wave a magic wand or if there were unlimited funds, there needs to be a structure starting from primary care that offers lifestyle support in a definitive way. A lot of people don't engage with the information that's out there.” (P7) “They don't seem to know what α_1_ is. I had to explain it to them myself.” (P2) “Originally, the GP diagnosed asthma, but the medications weren't working. It took about 8 years before the test was done, and by then, my health had deteriorated.” (P5) “Healthcare needs to get a grip on the fact that support should start at the level of primary care. Clinics or other methods need to offer direct, step-by-step advice in line with how severe the disease is, to help prevent the problems before they escalate.” (P7)	“The GP will only recognise the referral route to PR if they've actually got COPD.” (HCP6) “I think it's important to diagnose and then understand their management. I don't think a consultant can manage everything about a patient with α_1_ because obviously, they've got multiple different problems related to their systemic side of it. The consultant is under a huge amount of pressure to diagnose, manage and then hand back. People should be managed within their general practice in terms of their health. The role of the consultant is in diagnostics and optimisation, but they have to hand back to the GP, and it's up to the GP to then know they've got somebody with α_1_ and read up on what that patient needs locally. Pulmonary rehab needs to be locally delivered.” (HCP1) “We have to remember that they might be emphysema and not COPD… someone with α_1_ could be breathless with emphysema but not have obstructive spirometry, and that's a difficult cohort because they probably would benefit even though they're not classified as COPD.” (HCP1) “Those conversations are really… the most important part of the conversation is having somebody that's informed generally that knows where their local courses are, who it's by, what the parking's like, or the transport.” (HCP2)
**Emotional and social support**	“I felt out of place in a group full of COPD patients… It's just different for us.” (P3) “Meeting others with α_1_ would make it less lonely, knowing others understand.” (P5) “It was mentally tough, really tough for me. On my first trip there, I ended up in tears. It was hard to face what I couldn't do anymore, having always been fit and active.” (P11) “Oh, it is to know that you're not alone. That was the biggest boost to me and also to my wife.” (P7)	“Support groups for those with similar conditions may offer a solution to reduce feelings of isolation.” (HCP3) “One AATD patient's feedback even inspired the creation of a ‘Keep Moving’ booklet to encourage continuous activity after rehab. It was a simple idea but made a big difference in helping them stay motivated.” (HCP2) “One thing that resonates with me regarding AATD patients is the frustration and anger some feel when diagnosed with COPD caused by smoking, especially when they're non-smokers.” (HCP4) “In my experience, AATD patients tend to be on the more severe end of the spectrum by the time they reach rehab, often with higher oxygen requirements.” (HCP9)
**Post-rehabilitation support**	“I had a passport to a leisure card, and I went to the gym. But when I got there, the guy who was supposed to help wasn't around, and when he was, he didn't seem to want to help much. I felt lost, to be honest.” (P10) “When I did the rehab, they wrote out the programme for me every week, increasing the exercises and intensity. That was brilliant because I knew exactly what to do. But once I was discharged, I didn't have that kind of structure anymore.” (P12)	“Many services used to run a maintenance programme where patients could continue attending once a week. However, due to costs, funding for that stopped.” (HCP3) “The biggest issue is the lack of accountability once patients finish PR. During PR, there's someone checking if they attend, but after PR, no one is monitoring them.” (HCP2) “Patients often receive personalised programmes to continue after PR, but without the same accountability and feedback mechanism they had during the in-person service. This is an area that could be improved.” (HCP5)
**Technological integration**	“It's great because I push myself when I'm doing it [Zoom-based PR], which is good and it's so easy.” (P4) “I think, for these activity apps, they're done for people who are fit, they're done for people who are keeping fit, they're done for athletes. They're not done for people who sit.” (P11)	“There's potential for fitness apps, especially for budget-conscious patients, to follow a routine at a basic gym or at home.” (HCP3) “Some remote programmes fail because they don't do proper assessments before and after. It's crucial to understand a patient's fitness level and capacity before starting.” (HCP1) “Online rehab was well-received by some who couldn't attend due to location or schedule conflicts. It removed barriers for these patients, but face-to-face rehab should still be the primary option.” (HCP4)

Patient quotes illustrate lived experiences, barriers and facilitators related to PR accessibility, customisation and post-rehabilitation support. HCP quotes provide clinical perspectives on service provision, challenges in implementing personalised PR approaches and potential improvements to referral pathways, emotional and social support mechanisms and technological integration.

**FIGURE 1:**
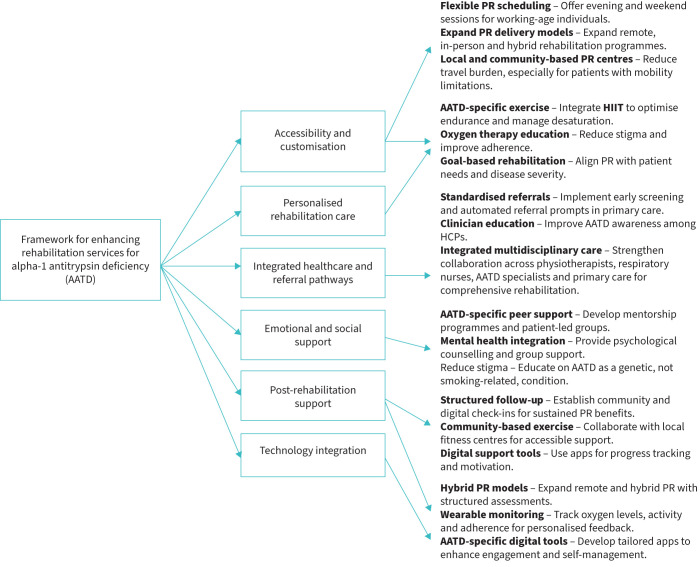
Critical-to-quality tree diagram illustrating key areas for optimising pulmonary rehabilitation (PR) services for patients with α_1_-antitrypsin deficiency (AATD). The diagram categorises key improvement areas into six main themes: accessibility and customisation, personalised rehabilitation care, integrated healthcare and referral pathways, emotional and social support, post-rehabilitation support and technology integration. Each theme includes specific needs, quality drivers and performance requirements identified from qualitative insights. Created using Lucidchart. HIIT: high-intensity interval training; HCP, healthcare professional.

Figure 1 presents the CTQ diagram, developed from final framework matrix outputs and team discussion. It categorises key findings into six domains and visually maps the quality drivers and performance requirements identified by participants. The CTQ framework translates thematic insights into actionable improvement targets for tailoring PR to those with AATD.

### Theme 1: accessibility and customisation

This theme reflects the need to improve access to and personalisation of PR by addressing geographic, scheduling and logistical barriers. Patients and HCPs emphasised that access to local PR services is critical for improving participation. Several participants cited long travel distances as a barrier, with one patient (P3) stating, “It would make a big difference to have somewhere close by.” HCPs echoed this concern; HCP2 noted, “Patients would likely prefer to go to a centre closer… rather than travel far.” Participants perceived that poorly located programmes may be underutilised.

Work and family commitments further restricted PR attendance, particularly among younger patients. P5 reflected, “I couldn't keep up with regular times because of work”, while HCP3 shared that some patients were referred to community-based services with flexible hours to improve engagement. Participants advocated for more localised and flexible PR delivery, such as evening or weekend sessions, as a strategy to improve adherence and reduce dropout.

### Theme 2: personalised rehabilitation care

Participants emphasised the value of goal-directed, individualised rehabilitation tailored to AATD-specific challenges, particularly exertional desaturation. P8 described their approach: “α_1_ patients… should do as much as they can. When you've done enough, you should try and do a bit more.” HCP4 reinforced this, stating, “We base our exercises on patients’ goals, making them very goal-focused.”

HIIT was commonly discussed by HCPs as a strategy for managing desaturation while improving aerobic capacity. HCP5 noted, “For individuals who desaturate, HIIT is a useful way of prescribing aerobic exercise.” Some patients also described difficulty with oxygen levels during exertion; P9 noted, “Once I start exercising, my oxygen saturation drops right down to 80 quite quickly.”

The role of oxygen therapy was debated. While HCPs highlighted its potential to support activity, patients often described stigma and reluctance. As HCP2 explained, “Many are reluctant to use portable oxygen because they don't want to be seen with it, leading them to do less.” These insights suggest the importance of individualised PR plans that integrate goal-setting, oxygen education and stigma-reduction strategies.

### Theme 3: enhancing diagnosis and referral pathways

Participants frequently described delays in diagnosis and confusion around PR referral, particularly at the primary care level. Several patients described misdiagnoses or long delays before appropriate testing. P2 said, “It took about 8 years before the test was done, and by then my health had deteriorated.” P6 similarly noted, “The GP… doesn't seem to know much about α_1_.” HCP7 acknowledged the challenge: “In my 13 years as a physiotherapist, I've seen about 11 patients… it's not common, so not everyone has experience with it.”

To support earlier referral, HCP8 suggested providing quick-access decision tools: “It might be nice to signpost patients to some info or contact details… quick, friendly resources would help if clinicians wanted to ring a consultant or email for further advice.”

Participants also emphasised the need for streamlined and clearer referral pathways. P5 remarked, “Healthcare needs to get a grip… start at primary care with direct advice.” HCP6 added that some AATD patients may not meet traditional spirometry criteria for COPD yet would still benefit from PR. These findings point to opportunities for improved clinician education and structured pathways that support earlier intervention.

### Theme 4: emotional and social support

Many patients described feelings of isolation or disconnection from standard COPD PR groups. P5 shared, “Meeting others with α_1_ would make it less lonely, knowing others understand”, while P3 recalled, “I felt out of place in a group full of COPD patients… It's just different for us.”

HCPs similarly acknowledged that while peer support is beneficial, there are few formal AATD-specific groups available. HCP1 stated, “Support groups are great, but there aren't enough of them.” Participants saw value in structured peer support tailored to the AATD experience, both for emotional well-being and sustained PR engagement. Stigma around genetic COPD and perceived blame was also discussed as a unique psychosocial barrier.

### Theme 5: post-rehabilitation support

Participants widely reported that follow-up after PR discharge was inconsistent or absent, limiting their ability to maintain physical activity. P10 noted, “I had a leisure card, but when I got to the gym, no one was there to help, and I felt lost.” Others recalled benefits from structured support during PR that was not sustained afterward.

HCPs described service-level constraints. HCP3 said, “Many services used to run maintenance programmes, but funding for that stopped.” Some suggested community-based follow-ups or periodic check-ins as alternatives. Participants emphasised the value of continued guidance and accountability, which could be delivered through in-person or digital mechanisms.

### Theme 6: technology integration

Participants identified remote and hybrid PR models as particularly helpful when facing travel-, time- or health-related barriers. P4 explained, “Zoom-based PR is great because I push myself when I'm doing it, and it's so easy to join.” However, HCP8 cautioned that structured assessment before and after remote delivery is needed to ensure effectiveness.

Participants also noted the lack of tailored digital tools for AATD self-management. P9 remarked, “I've not come across an app yet that says, ‘This is your app. You've got α_1_.’ ” Both patients and HCPs viewed customised technology as a potential avenue to support engagement, especially where long-term follow-up was otherwise limited.

## Discussion

This study offers novel qualitative insights into the perspectives of patients with AATD and HCPs regarding PR. The findings highlight opportunities to improve PR engagement and effectiveness by addressing known barriers such as accessibility, personalisation and psychosocial support while also uncovering AATD-specific challenges. These findings extend and reinforce previous work, such as the European Alpha-1 Research Collaboration (EARCO) survey, which emphasised diagnostic delays, lack of tailored services and the need for improved self-management strategies in AATD care [6, 18].

By integrating patient and HCP perspectives, this study provides a holistic view of real-world service delivery gaps and generates actionable recommendations, many of which are mapped within a CTQ improvement framework. While consistent with prior COPD and rare disease literature [6, 11, 14], this study contributes new understanding of how these challenges manifest for AATD and which adaptations may be most impactful.

### Key findings and interpretation

#### Barriers to PR accessibility and participation

Travel distances and work or caregiving responsibilities commonly disrupted PR attendance. These findings echo existing COPD literature [4, 7, 13, 22], but have amplified relevance in AATD, which typically affects younger adults. Participants highlighted the value of localised or hybrid PR programmes offered outside standard working hours to improve flexibility and uptake. Prior studies have shown that hybrid PR can maintain clinical benefits while increasing reach [23]. Early intervention in AATD is critical given the risk of rapid lung function decline [2, 14], and expanded community- or home-based options may enhance long-term engagement [13, 23, 24].

#### Personalised rehabilitation and disease-specific strategies

Participants strongly endorsed the value of tailored exercise prescriptions, particularly in the context of exertional desaturation. HIIT was viewed by HCPs as a practical approach to balance effort with oxygen management, though evidence specific to AATD remains limited [8, 11, 12]. While HIIT improves dyspnoea and functional capacity in COPD [10, 15], its impact in AATD has not been rigorously evaluated. Thus, while frequently discussed, HIIT and oxygen therapy cannot yet be considered “central” to PR in AATD; rather, they represent promising, underexplored strategies [10, 13].

Patients expressed reluctance to use supplemental oxygen in public settings, citing stigma and inconvenience, concerns supported by prior research [14, 16, 17]. Integrating oxygen education and addressing social stigma directly in PR delivery may improve adherence and confidence [8, 10, 14]. These findings align with previous physiological and epidemiological research on exercise responses, screening and disease burden in AATD [25–30].

#### Delayed diagnosis and referral barriers

Consistent with EARCO findings and previous studies [6, 18, 20], participants described long delays before AATD was diagnosed often due to limited primary care awareness. As one patient described, symptoms were misattributed to asthma for years. Such delays reduce the window for preventive intervention and referral to PR. Interventions such as routine screening prompts in electronic health records, brief primary care education modules and referral flow charts may expedite diagnosis and rehabilitation access [6, 14, 22].

#### Psychosocial barriers and peer support needs

Participants frequently reported feeling disconnected or “different” in conventional COPD PR groups. These insights align with previous research indicating that AATD carries a distinct psychosocial burden, including feelings of isolation, difference and internalised stigma, particularly when engaging with peers whose condition is related to smoking [13, 22]. Structured AATD-specific peer support delivered through support groups or forums, or integrated into PR, may help normalise the experience and reduce emotional barriers to participation [4, 13, 22, 31, 32]. While organisations such as the Alpha-1 Foundation (USA) and Alpha-1 UK Support Group provide valuable resources, access remains variable across regions.

#### Post-rehabilitation support and long-term engagement

Sustaining gains after PR discharge remains a common challenge across respiratory conditions. Participants reported a lack of structured follow-up, which limited motivation and continuity. HCPs also noted the decommissioning of maintenance programmes due to funding cuts. Prior COPD studies support the role of community-based exercise, remote follow-ups and patient-held plans in maintaining benefits [23, 24, 33]. In this study, participants valued low-cost, personalised resources such as printed exercise guides and online tools, emphasising the need for scalable, adaptable long-term models.

#### Study strengths and limitations

This is the first qualitative study to explore PR optimisation specifically for AATD by integrating patient and clinician perspectives [11, 14, 18]. The use of the Framework Method [21] ensured systematic, transparent analysis, and triangulation strengthened the credibility of findings. In addition, developing a CTQ diagram provides a visual, actionable synthesis of improvement priorities, which may be valuable for service designers.

Limitations include the single-centre design and lack of functional outcome data, which limit generalisability. The sample also lacked ethnic and socioeconomic diversity, which may constrain transferability to more diverse populations or underserved regions [6, 13, 18]. For instance, experiences of stigma or access to hybrid PR models may vary in minoritised communities or in areas with limited digital infrastructure. Future studies should prioritise inclusive recruitment and explore how sociodemographic factors influence PR access and outcomes in AATD [11, 14].

#### Implications for practice

While some barriers identified in this study are shared with other chronic respiratory diseases (*e.g.*, COPD), others, such as younger patient age, genetic stigma and diagnostic delay, highlight the need for adapted, rather than entirely distinct, PR models. Tailored modifications may include embedding AATD-specific peer support into existing PR programmes [13, 31, 32], offering flexible scheduling and hybrid delivery models [23] and incorporating HIIT protocols to accommodate rapid desaturation [10, 11, 15]. Implementation could involve targeted staff training [8], integration of referral prompts into electronic health records [7, 22] and use of low-cost digital tools for long-term follow-up and remote monitoring [23, 24, 33]. However, practical barriers such as variable workforce capacity, service funding and digital inequities may limit uptake, particularly in underserved or digitally excluded populations [6, 13, 34]. Addressing these challenges will require coordinated efforts in workforce development, inclusive digital infrastructure and service co-design with patients and frontline providers [7, 8, 11, 14].

### Conclusion

This study highlights the need for tailored, patient-centred PR models to address the clinical and psychosocial challenges of AATD. Key improvement areas include flexible delivery, personalised exercise for symptom variability and clearer referral pathways. Findings support adapting existing PR frameworks rather than creating separate services. The CTQ diagram offers a practical tool for service design. Further research should evaluate hybrid models and sustained support strategies.

## Supplementary material

**Please note:** supplementary material is not edited by the Editorial Office, and is uploaded as it has been supplied by the author. Supplementary material 00332-2025.SUPPLEMENT
