# Supplementary material for: Optimising pulmonary rehabilitation for α1-antitrypsin deficiency: a qualitative study of patient and clinician perspectives
Source: ERJ Open Res. 2026 Mar 9;12(2):00332-2025. doi: 10.1183/23120541.00332-2025 (PMC12969663; doi:10.1183/23120541.00332-2025)
Supplement: Supplementary file 1 [file 00332-2025.SUPPLEMENT.pdf]

OPTIMIZING REHABILITATION FOR CHRONIC OBSTRUCTIVE PULMONARY DISEASES PATINETS WITH UNDELYING ALPHA-1 ANTITRYPSIN DEFICIENCY (AATD)

Patients Intervew Topic Guide

General knowledge about alpha Alpha-1 Antitrypsin Deficiency (AATD)

- Describe you basic understating about the disease Alpha-1 Antitrypsin Deficiency

Explore

- How is the alpha one affecting your general health?

Wellbeing – vitality – physical activities

- What are your health concerns being diagnosed with alpha one?

Prognosis of the disease – impact of the disease on health

- How do you overcome these concerns?

Support group – health care counselling

- What is your best source of information about alpha one?

Online – physicians – GP

- How often do you monitor your general health?

annual physical examination – routine health checks up

- How are your relatives encouraged to do AATD screening?
- What lifestyle modifications do you think are important for alpha one patient?

things to do, not to do.

- What do you think about peer support in disease management?

Network with patients with similar diagnosis

- What do you recommend improving disease management for alpha 1 patient?

Government support –health care service modification –

Previous experience with pulmonary rehabilitation program

- Describe your previous experience with the Pulmonary Rehabilitation program

  Explore

  - What was the impact of the program on your quality of life?

vitality, energy, social functioning, quality of sleep, disease exacerbation, physical activities etc.

  - How did the program change **your attitude toward exercise**?
  - How **would you describe the accessibility of the program**?

referral – appointment – wait time – setting?

  - Did **you find the exercise program challenging for you**?

Exercise intensity – equipment – format of the exercise session, type of exercise

  - Do **you find yourself motivated to continue with the home-based exercise recommendations**?

Current Exercise routine – what sort of recommendation do you receive

  - How **to improve adherence to routine exercise after the program**?

  - What **do you think the advantages an disadvantages of remote delivery of the program**?

Home based pulmonary rehabilitation

  - Do you think you have received enough education about alpha one as part of your disease management?

Education topics – interesting knowledge – what do you know about the alpha one?

  - What are your long-term goals for exercise and physical activates?
  - What improvement do you suggest for a pulmonary rehabilitation program specific for AATD patients?

Attitude toward exercise and physical activities

- Describe your attitude toward exercise and physical activities

  Explore

  - What is your **motivation** to exercise?
  - What **kind of exercises and activates** do you like to do?

- Where **do you prefer to exercise**?

Home- local gym – parks – community centers

- How many days per week do you aim to exercise? Why?
- What are **the barriers to** engage in routine exercise?

Time – energy – other diseases

- **What type of exercise do you prefer**?

Examples: **strength exercise** (TheraBand exercise, dumbbell exercise, resistance exercises) **Aerobic exercise** (treadmill walking, stationary bikes, jogging, running), or Combination of strength and Aerobic exercise., **Yoga**

- **How do you monitor your exercise routine**?

Exercise diary – exercise monitoring – activity tracker

- **What do you think about the use of technologies in guiding exercise activities**?

(Wearables, virtual contents, phone apps)

- **What do you think the barriers for technology usage**?
- What **are common symptoms associated with physical activities and exercise if any**?

breathlessness, tiredness, rapid breathing etc.

- How do **you overcome challenges associated with** exercising and physical activities?

Is there anything else about exercise and your health that you would like to tell me? is there anything else you would like to talk about…..

Topic Guide – Health Care professional HCPs

Healthcare Professional Knowledge about AATD:

Explore:

Expansion of PR services for AATD and wider respiratory conditions

- General experience and Knowledge about AATD
- Previous Experience with AATD Patients
- Adequacy of AATD Education Received: Impact of Staff Knowledge on Patient Satisfaction
- Ask for examples.

Challenges in Program Delivery:

Explore

a. Initial Patients' Assessment for PR

- Common Expectation About PR Services among AATD patients
- Discussion on AATD Diagnosis during initial assessment
- Approach to addressing psychological problem among patients.
- Process of Goal Setting? Does it differ for AATD
- Criteria for Declining Access to PR? Idea of too fit for rehab, given the current structure of the program

b. During Program Delivery:

- What is the best exercise prescription for these patients based on your experience? An why?
- Adapting Exercise Choices Based on Research Findings: example: AATD had faster exercise O2 desaturation, how this could change exercise choices for them?
- Strategies to Prevent Isolation among cohort of different age, and physical abilities.
- Overcoming Patient Intimidation of exercise types and intensity

c. PR Graduates: Post-PR Completion:

- Many AATD patinets were keen to maintain exercise after discharge, what is your experience?
- What is the current mechanism to encourage that?

- Thoughts on Incorporation of Technology in PR Programs (e.g., Telemedicine, Remote Monitoring)
- Mechanisms for Post-Discharge Communication: for patients to communicate their challenges and success post PR.

PR Educational Topics:

- Balancing General Education with Condition-Specific Information
- Need for Targeted Education for AATD Patients (e.g., Support Groups, Nutrition Advice)
- Innovative Educational Tools or Methods
- Mechanisms in place to Evaluate Educational Topic Efficacy

Is there anything else we did not cover that you would like to tell me about….

End….
